# Endocannabinoid Signaling for GABAergic-Microglia (Mis)Communication in the Brain Aging

**DOI:** 10.3389/fnins.2020.606808

**Published:** 2021-02-03

**Authors:** Jorge Carrera, Jensen Tomberlin, John Kurtz, Eda Karakaya, Mehmet Bostanciklioglu, Onder Albayram

**Affiliations:** ^1^Division of Cardiology, Department of Medicine, Medical University of South Carolina, Charleston, SC, United States; ^2^Department of Neuroscience, Medical University of South Carolina, Charleston, SC, United States; ^3^Elysium Health Center, Gaziantep, Turkey; ^4^Ralph H. Johnson VA Medical Center, Charleston, SC, United States

**Keywords:** GABAergic neuron, microglia, neuroinflammantion, brain aging, endocannabinoid system

## Abstract

The aging brain seems to be characterized by neuronal loss leading to cognitive decline and progressively worsening symptoms related to neurodegeneration. Also, pro-inflammatory states, if prolonged, may increase neuronal vulnerability via excessive activation of microglia and their pro-inflammatory by-products, which is seen as individuals increase in age. Consequently, microglial activity is tightly regulated by neuron-microglia communications. The endocannabinoid system (ECS) is emerging as a regulator of microglia and the neuronal-microglia communication system. Recently, it has been demonstrated that cannabinoid 1 (CB1) receptor signaling on GABAergic interneurons plays a crucial role in regulating microglial activity. Interestingly, if endocannabinoid signaling on GABAergic neurons are disturbed, the phenotypes mimic central nervous system insult models by activating microglia and leading to accelerated brain aging. Investigating the endocannabinoid receptors, ligands, and genetic deletions yields the potential to understand the communication system and mechanism by which the ECS regulates glial cells and aspects of aging. While there remains much to discover with the ECS, the information gathered and identified already could lead to the development of cell-specific therapeutic interventions that help in reducing the effects of age-related pro-inflammatory states and neurodegeneration.

## Introduction

Aging is the primary factor in the rise of neurodegenerative disorders because of accumulation of mitochondria dysfunction and disrupted intercellular communications (Hou et al., 2019). Neurons are post-mitotic cells making them particularly susceptible to the age-related dysfunctions that will cumulate in progressive decrease in brain functions related to pro-inflammatory and pro-oxidative states (Saez-Atienzar and Masliah, 2020). Mitophagy, inflammatory states, and metabolism are primarily mediated by microglia in the brain to maintain normal neuronal viability and communications (Wolf et al., 2017; Valles et al., 2019; Vainchtein and Molofsky, 2020). The problem with neurodegenerative diseases is that each person has individual variability of neurodegenerative symptoms, but it is not known whether the variability stems from pathological causes or age-related changes in the brain. The confusion is best illustrated by the knowledge that most aged persons will experience mild physiological decreased brain performance; whereas a few individuals will show increased cognitive impairment and suffer from neurodegenerative disorders such as Alzheimer’s disease. At the moment, the age-related changes that affect brain function lead to altered synaptic plasticity and neuronal connectivity (Bano et al., 2011). The downstream effect is alterations in CNS cellular communication that leads to neuronal vulnerability, cognitive decline, and neurodegenerative disorders from chronic pro-inflammatory states via the CNS innate immune response (Bilbo et al., 2012; Cribbs et al., 2012; Hou et al., 2019).

Microglia are the CNS immune cells that continuously survey the brain parenchyma and rapidly respond to changes in the brain (Nimmerjahn et al., 2005). In response to pro-inflammatory cytokines and other signaling molecules, they can transition to an active state by altering glial-specific intermediate filament proteins in the cytoplasm, resulting in extended cellular processes, diminished cellular processes, and soma hypertrophy (Sierra et al., 2007). In the normal healthy brain, activation of microglia is a complex process requiring well-organized and tightly regulated communications with neurons and extrinsic factors (Wendeln et al., 2018; Valles et al., 2019; Salas et al., 2020). As such, the study of microglia regulation and communication is crucial to derive new therapies and develop knowledge of neuropathies related to aging. The endocannabinoid system (ECS) is emerging as a neuroprotective system by regulating age-related effects of chronically activated microglia. Current evidence shows the ECS directly effects microglia or indirectly alters the neuron-microglia communication system leading to a reduction in oxidative stress and increased clearance of damaged macromolecules (Bilkei-Gorzo, 2012). With their relation to neuronal and microglial pro-inflammatory by-products, the ECS is an ideal candidate for study in relation to the neuron-microglia communication system and future therapeutics, since the ECS acts as a feedback communication route that modulates the secretion of neurotransmitters from the presynaptic terminals of neurons that may affect microglia (Basavarajappa et al., 2017; Cristino et al., 2020).

## The Endocannabinoid System and Changes During Brain Aging

**FIGURE 1:**
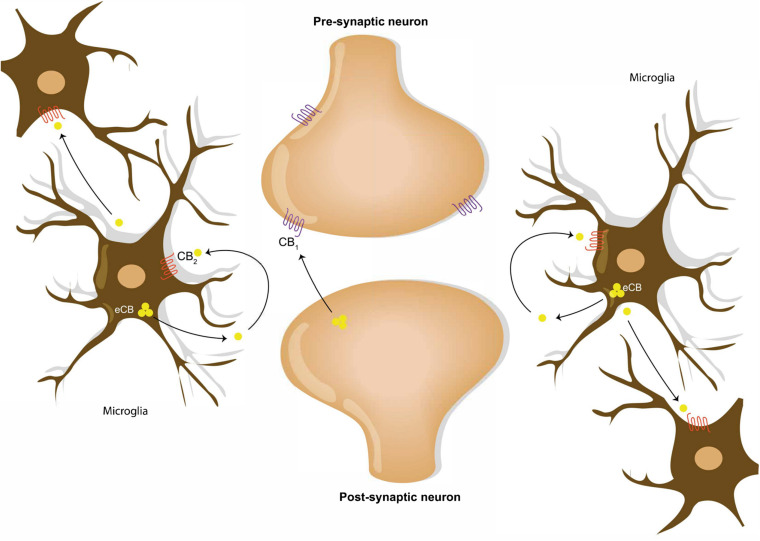
Endocannabinoid signaling in neural and microglial circuits. Retrograde neuronal endocannabinoid signaling refers to the process of the release and the travel of endocannabinoids backward from the postsynaptic neuron to bind to the CB1 receptors on the axon terminal of a presynaptic neuron. Microglial cells communicate via release of endocannabinoid into the extracellular space, which bind to the CB2 receptors on microglia cell membranes.

Throughout the last decade, the extensive research into endocannabinoids suggest that the system may play a new and vital role in the regulation of healthy brain aging. The ECS is comprised of endogenous cannabinoid receptor ligands, biosynthetic and metabolic enzymes, and G-protein coupled cannabinoid type 1 receptor (CB1) and cannabinoid type 2 receptor (CB2) expressed in neuron and microglia, respectively (Luongo et al., 2010; Albayram et al., 2011; Figure 1). In a “normal” and healthy individual, CB1 receptors are the most abundant GPCR in the body and is localized in the throughout the brain, but it is predominately localized in neurons (Di Marzo et al., 2015). Histologic studies have determined that CB1 receptors in the hippocampus and the cortex are highly expressed GABAergic neurons, specifically 90% of cholecystokinin -positive and 10% of calbindin-positive interneurons (Katona et al., 1999; Marsicano and Lutz, 1999). The majority of other GABAergic neurons are negative for CB1 receptors (Katona et al., 1999). As for CB2 receptors, the basal CNS level exists as trace amounts among neurons and glial cells (Stella, 2010; Di Marzo et al., 2015). In response to a CNS insult and other pro-inflammatory states—such as aging—it is the microglia that drastically upregulate expression of CB2 receptors and the synthesis of endocannabinoid ligands (Stella, 2010; Di Marzo et al., 2015). Under “normal” circumstances, the ECS provides a feedback mechanism by which microglia respond to presynaptic signals originating from neurons by releasing endocannabinoids in an attempt to inhibit GABAergic signaling (Alger, 2002). The dominant and most studied endogenous cannabinoid receptor ligands are arachidonoylethanolamide (AEA) and 2-arachidonoylglycerol (2-AG). While both ligands are agonist, AEA—the first discovered ligand—has low-efficacy and high affinity for CB1 receptors with even less efficacy for CB2 receptors unlike 2-AG that has lower affinity but is equally effective on CB1 and CB2 receptors (Luongo et al., 2010; Di Marzo et al., 2015). The endocannabinoid ligands are produced on demand—synthesized as necessary and metabolized once activating signals end (Ativie et al., 2018; Bilkei-Gorzo et al., 2018). The synthesis of the ligands is dependent on diacylglycerol lipase α (DAGLα); whereas, monoacylglycerol lipases (MAG) and fatty acid amide hydrolases (FAAH) are involved in their metabolism (Di Marzo, 2011). Evidence shows that astrocytes and microglia possess the enzymatic potential to catabolize and anabolize endogenous cannabinoid receptor ligands in high quantities (Carrier et al., 2004; Walter et al., 2004; Muccioli et al., 2007). In fact, it is a known that microglia exceed neuron and astrocyte product of 2-AG by twenty times (Walter et al., 2003). However, the concentration of receptors and the expression endocannabinoids depends on the brain area, the type of cell, and the inflammatory state of the CNS (Klein, 2005). In the hippocampus, an area of focus for the aging research field because of its changes in memory, morphology and electrophysiological properties in older subjects (Erickson et al., 2012), signaling via the ECS might influence the expression and activity of three membrane bound ligand-receptor pairs associated with immune response in hippocampal neurons and glia (Albayram et al., 2011).

**FIGURE 2:**
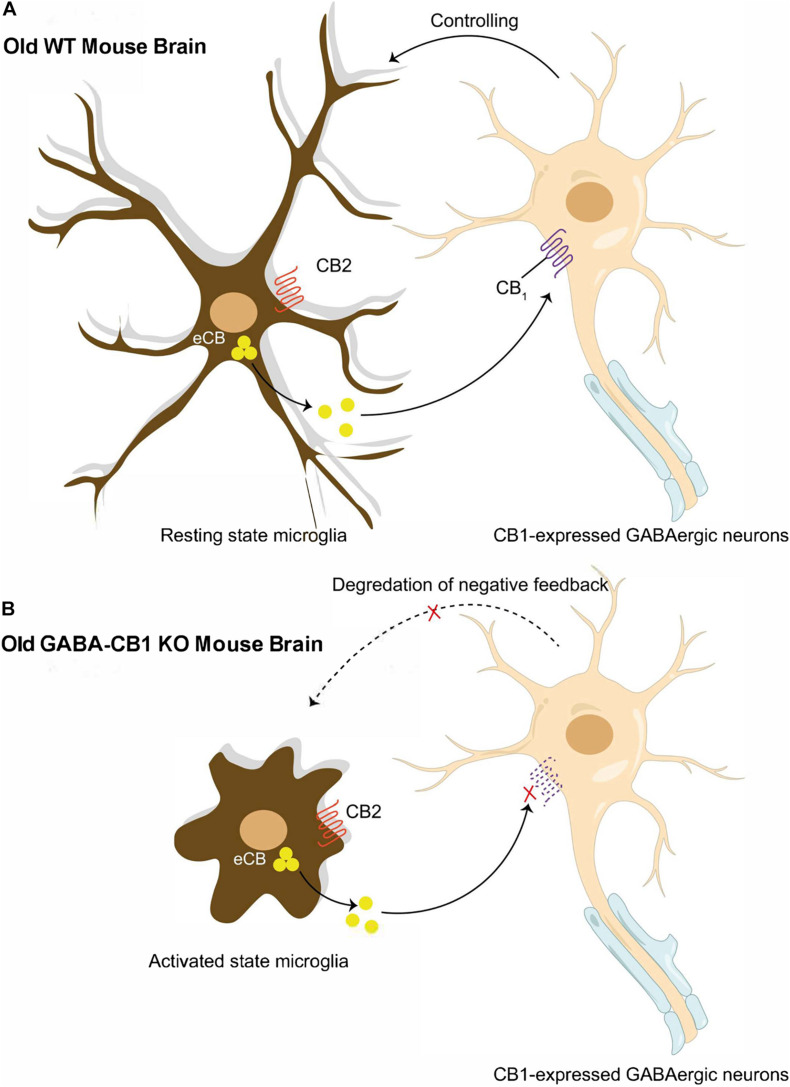
Reciprocal interactions between microglia and presynaptic GABAergic neuron. **(A)** Old WT mouse brain: Normal neuronal-glial communication prevents microglial reactivation through endocannabinoid signaling between CB1 expressed GABAergic neurons and microglia. **(B)** Old GABA-CB1KO mouse brain: Disrupted endocannabinoid signaling between CB1 expressed GABAergic neurons and microglia break down control of microglial reactivation and thus, exacerbating age-dependent neuro-inflammation in the brain.

As research in the ECS continues, it has become abundantly clear that there is age-related alteration in ECS formation and function that can itself accelerate aging through synaptic dysfunction and impaired plasticity. *In vitro* studies have shown ECS modulates reactive oxidative species (ROS) formation through 2-AG with the hypothesized mechanism being attenuation of mitochondrial oxidative phosphorylation via decreased oxygen consumption (Bilkei-Gorzo, 2012). The ECS is also responsible for an increase in brain-derived neurotrophic factor (BDNF) that declines with decreased neurogenesis in a genetic deletion of CB1 receptors (Albayram et al., 2011; Bilkei-Gorzo, 2012). The system itself experiences age-related decline. In rodent histological samples, there are significant declines in CB1 receptor mRNA and agonist binding—particularly in the hippocampus (Bilkei-Gorzo, 2012; Di Marzo et al., 2015). Similar results were seen in human histological samples (Bilkei-Gorzo, 2012). With modulations in age-related process, genetic deletions of CB1 receptors or Cnr1 (the gene that codes for CB1 receptors) knockout mice were used to confirm hallmark signs of accelerated aging. The use of old CB1−/− mice and Crn1−/− knockout (KO) mice showed decreased production of AEA, increased neuronal loss, the presence of chronic pro-inflammatory states, and impairment in learning and memory skills compared to age-matched wildtype mice–changes which were particularly noticed and verified in hippocampal GABAergic neurons using a GABA-Cnr1−/− KO mouse model (Albayram et al., 2011; Bilkei-Gorzo, 2012; Di Marzo et al., 2015; Ativie et al., 2018; Figure 2). The CB2 receptor, compared to the CB1 receptor, has been far less researched; however, a CB2−/− receptor mouse model showed decreased responsiveness to pro-inflammatory stimuli and decreased microgliosis that may play a role in the age-accelerating affects when discussing ECS and glial regulation (Chen et al., 2017).

## Neuronal-Microglial Communication

In a healthy individual, microglial are in a resting state that continuously surveys the entire brain parenchyma (Nimmerjahn et al., 2005), and upon encountering an activating trigger via the TLR or IFN-γ pathways, microglia gradient transition into ameboid is the “activated” state that responds to CNS injury (Kettenmann et al., 2011; Colonna and Butovsky, 2017). The phenotype and the microglial subtype that undergoes gliosis depends on pro-inflammatory signaling molecules of the surrounding damaged tissue, as well as signals from neurons via soluble factors (Pocock and Kettenmann, 2007) and direct cell-to-cell surface interactions (Hoek et al., 2000; Cardona et al., 2006; Bessis et al., 2007). Natural disturbances accumulate throughout aging resulting in more severe and prolonged pro-inflammatory states that contributes to symptoms associated to neurodegeneration (Hanisch and Kettenmann, 2007). In this sense, the CNS immune system has dual contrasting roles that are primary managed through the neuron-microglia communication system.

Microglia support neurons and have receptors for neurotransmitters, and neurons are responsible for inhibitory control of leading to examine the communication system between the two cell types (Chavarria and Cardenas, 2013). In microglia, the bi-directional communication between neurons and microglia is through adhesive and secreted forms of chemokines, such as the fractalkine (CX_3_CL1), the CD200 signaling axis, activation of purinergic receptors (P2Y12R), neurotransmitters, and extracellular vesicles (EVs). The CX_3_CR1 receptor is located on microglia, and the CX_3_CL1 ligand is located on neuronal membranes where it regulates microglia (Ativie et al., 2018). Notably, a CX_3_CL1 receptor knockout was shown to reduce pro-inflammatory cytokines and attenuated microglial activation in wildtype aged rats following lipopolysaccharide (LPS) injection (Lyons et al., 2009). CD200 is an attached and soluble ligand located on neurons, astrocytes, and oligodendrocytes with its receptor CD200R located exclusively on microglia (Barclay et al., 2002), and interactions between the ligand and receptor promotes a “resting” microglial phenotype and a decrease of pro-inflammatory cytokines (Hoek et al., 2000; Banerjee and Dick, 2004; Cox et al., 2012). As for the microglial-P2Y12R, the key feature is the ability to promote chemotaxis and motility of microglial architecture or to sense ATP at sites of acute brain injury to promote microglial neuroprotection (Eyo et al., 2017, 2018). When the microglia reach the site of injury, the microglia can form somatic junctions with the neuron to enhance neuronal mitochondria and the release of neuronal ATP to increase microglia coverage (Cserep et al., 2020); however, these junctions can form in healthy tissue leading to neuronal regulation through neurodevelopment and neurogenesis, microglial ATP-mediated and P2Y12R-dependant alterations in excitatory postsynaptic potentials (EPSPs) through a microglial negative feedback system that converts neuronal ATP to a neurosuppresive metabolite, or increased neuronal excitability with a P2Y12R KO mouse model (Eyo and Wu, 2013; Mo et al., 2019; Peng et al., 2019; Badimon et al., 2020). The state of the neuron excitability and injury dictates the microglia response based on ATP but not calcium for the P2Y12R. Hyperactive and hypoactive neurons can both create calcium influx via the P2Y12R leading to increase microglia process outgrowths (Eyo et al., 2014; Umpierre et al., 2020). The P2Y12R is of special importance because it is sensitive to changes in GABA—when levels of GABA transmission drop, then there is an increase of microglia chemotaxis and motility to once area (Eyo and Wu, 2013). GABA is not the only neurotransmitter to alter the affect the neuron-microglia communication system. Recently, studies of mice without anesthesia have seen the normal neuronal activity is coupled with increased levels of norepinephrine that has a suppressive effect on microglia function exclusively via the ß_2_-aderenergic receptor by decreasing arborization, motility, response to injury, dendritic connection, and effect on neuronal plasticity (Liu et al., 2019; Stowell et al., 2019; Hu et al., 2020). Similar to how neurotransmitters exert effects extracellularly, microglia EVs that affect both neurons and other microglia in separate pathways. Through direct action and EV cargo molecules, microglia may increase miniature excitatory postsynaptic current (mEPSC) neurotransmission, suppress neuronal apoptosis, and reduce dendritic spines in the hippocampus (Antonucci et al., 2012; Drago et al., 2017; Prada et al., 2018). The communication between neurons and microglia is essential to maintain homeostasis, which is why it is important in aging because disturbances may alter morphology and function of glia and disrupts their neuron-supportive roles.

Age-related changes affect microglia and evidence shows that aged microglia produce a slowed but exaggerated immune response coupled with reduced phagocytic potential, increased pro-inflammatory cytokine release, and increased ROS release that results in a longer immune response, a longer pro-inflammatory period, and enhanced neurotoxicity (Dilger and Johnson, 2008; Nakanishi and Wu, 2009; Luo et al., 2010; Jurgens and Johnson, 2012; Valles et al., 2019). Part of the exaggerated and altered immune response is derived from a more deleterious ameboid morphology witnessed in aged-microglia composed of fewer ramifications and branches that are smaller and more swollen (Hickman et al., 2013; Colonna and Butovsky, 2017). The slowed and sustained microglial immune response suggests the presence of immunosenescence, a process of age-related dysregulation of the brains immune system (Damani et al., 2011). The end result of the aging brain and aging microglia results in increased neuron vulnerability that accelerates neurodegeneration (Jurgens and Johnson, 2012).

## The Endocannabinoid System and Neuron-Microglia (MIS)Communication During Brain Aging

Several experiments have showed that retrograde communication via the ECS modulates a variety of functions including the regulation of glutamate excitotoxicity, altered proliferation and migration of the neural immune system cells, pro-inflammatory cytokine release, reduced ROS release, and lowered cerebral vasoconstriction (Walter et al., 2003; Carrier et al., 2004; Klein, 2005; Stelt and Marzo, 2005). The ECS mediates its effects via cannabinoid ligands, and microglia are a primary source of 2-AG—one of the main bodily endogenous cannabinoids—producing an amount that is twenty-fold higher than neurons and astrocytes (Walter et al., 2003). A surprising finding in light of the evidence is that microglia have barely detectable levels of ECS receptors at rest (Stella, 2010). When undergoing gliosis, however, CB2 receptors (and CB1 receptors to an extent) are upregulated in microglia opening the possibly for direct effects from the ECS to modulate its response. The typical function of endocannabinoids on CB1 and CB2 receptors on microglia are to counter pro-inflammatory mediators and retain normal phagocytic potential known to delay brain aging (Benito et al., 2008; Di Marzo et al., 2015; Plaza-Zabala et al., 2017). Endocannabinoid ligands in both the early and late phases of gliosis have been tested in rodent models (Schmidt et al., 2012) and the effects have been associated with prevention of neurodegenerative symptoms in disorders such as Huntington’s Disease (Ullrich et al., 2007; Palazuelos et al., 2009). Specifically, endocannabinoids have a direct effect on the interactions between microglial CD200 and CD200R via anandamide that enhances CD200R expression and increases IL-10 levels to have a neuroprotective effect (Hernangomez et al., 2012). In reciprocal communication, microglia may transmit AEA through EVs to stimulate CB1 receptors, leading to the suppression GABA inhibitory transmission that may lead to neuron vulnerability, if prolonged (Gabrielli et al., 2015). The same AEA transmitted via EVs can reach and activate other microglia to propagate the immune response (Szepesi et al., 2018).

Another line of evidence suggests that modulation of microglia regulation is not a direct effect through CB2 receptors but an indirect effect by CB1 receptor. Immediate effects of ECS communication inhibits transmitter release that can activate or inhibit neurotransmission depending on the type of neuron (Mechoulam and Parker, 2013); whereas, the long-term effects can elicit modification of gene transcription (Stelt and Marzo, 2005). The mediation of the indirect CB1 is primarily carried by GABAergic neurons of the hippocampus (Luongo et al., 2010). Specifically, approximately 90% of CB1 receptors are located on CCK + GABAergic neurons that are predominantly located in the ventral stratum lacunosum moleculare (slm) in the CA1 of the hippocampus, as supported by major differences in protein expression between WT and a CB1−/− receptor knockout mouse model in the slm (Jinno and Kosaka, 2006; Jinno et al., 2007). The high concentration of microglia and cholecystokinin (CCK)- expressing GABAergic neurons located in the ventral slm of the CA1 provides a strong basis for this layer and the ECS as an indirect modulating factor for neuron-microglia communications. The previous speculation is further confirmed by the fact that in the previously mentioned knockout mouse model only GABAergic neurons increase activation of microglia in aging—a non-observable effect in glutamatergic neurons (Albayram et al., 2011).

Evidence for the indirect microglia regulation of GABAergic CB1 receptors have been studied through miscommunication experiments with a GABA-CB1−/− knockout mouse model. This model is hypothesized to induce a modified expression of ligand-receptor pairs, which contributes to altered immune responses. As such, small perturbations of the signaling cascades leading to the expression of ligand receptor pairs could lead to a spontaneous activation of glial cells without any injury or infection (Kierdorf and Prinz, 2013). Therefore, efficient modulation of these molecules is essential in brain homeostasis, especially in terms of aging. Specifically, the CB1 receptors are a known point for glial regulation by neurons (Chavarria and Cardenas, 2013), so current research examines how the presence or absence of CB1 receptors affects neuron-glial communications and gliosis. Initial studies of a CB1—primarily controlled by GABAergic hippocampal neurons—null mice model identified that there was an increased level of microglia and pro-inflammatory cytokines in the molecular layer in wildtype mice (Cutando et al., 2013). Additionally, the microglia in this knockout show an altered reactivity to CNS insults and an altered wildtype morphology characterized by a larger cell body, increased ionized calcium-binding adapter molecule-1 (Iba1) density, and reduced number and complexity of branches (Ativie et al., 2018). As for the hippocampus, the lack of CB1 resulted in an accelerated loss of neurons in the CA1 and CA3 region that was associated with reduced efficiency in cognitive tests, specifically in the smaller population of GABAergic neurons (Albayram et al., 2012). One of the proposed mechanisms is the decreased levels of CX3CL—a neuronal control for microglia—in the knockout (Ativie et al., 2018). The slm region of the hippocampus is the primary site of alteration, indicated by increased positive areas, density, pro-inflammatory cytokines, and more detectable arborizations (Bilkei-Gorzo et al., 2018). In fact, the changes experienced by the lack of CB1 receptors are similar to a wildtype mouse injected with LPS (Ativie et al., 2018). As for CB2 receptors, current research has only looked at effects of the receptor on microglia. In a paradoxical result, the deletion of CB2 receptors in microglia also led to a reduction of the response to pro-inflammatory stimuli without a CNS insult, which is the same result as when wildtype mice are given a CB2 agonist (Schmole et al., 2015). Results apart from this aberration show that the deletion of the CB2 receptor also caused reduced gliosis, reduced pro-inflammatory cytokines, and reduced pro-inflammatory chemokines during a CNS insult, but it is clear that further research of this knockout model is required (Schmole et al., 2015). Given the research, the ECS is a key modulator for age-related symptoms such as pro-inflammatory changes can lead to neurodegenerative disorders, and the ECS is essential for proper communication between neurons and microglia making the communication system a potential target for neurodegenerative disorder therapeutics.

## Conclusion

Since glial cells have a strict arrangement in the hippocampal architecture in young mice, it is appealing to consider if this is the morphological equivalent of the functional units that glial processes form with the neurons they enfold. Current studies suggest that the ECS is an essential part of neuron and glial communication channel in the hippocampus that aids in neuroprotective events, reducing oxidative stress, regulation of glial activity, and clearance of damaged macromolecules. As such, the ECS activity and its disruption in aging is a potent model studying how neuron-glia communication is interrupted in normal brain aging, which may result in age-dependent neurodegenerative disorders. Normal communications between the neuron and glia through ECS activity tend to regulate neuroprotective and neurosupportive roles in glutamate excitotoxicity, proliferation, metabolic balance, pro-inflammatory cytokine release, and ROS release that all play a role in preventing neurodegeneration by a prolonged pro-inflammatory state. Currently, the direct mechanism for enhancing or inhibiting the pro-inflammatory state response is unknown, but there are still many aspects of the ECS to be researched to reduce or prevent accelerated age-related pro-inflammatory changes and brain aging. The current evidence provides a clear picture that the ECS is a crucial system for regulating neuron, glia, and the inflammatory process in the CNS that makes it a potential target for therapeutics due to its role in age-related pro-inflammatory states and brain aging.

## Author Contributions

All authors researched the data for the article, discussed the content of the article, wrote the text, and reviewed and edited the manuscript before submission.

## Conflict of Interest

The authors declare that the research was conducted in the absence of any commercial or financial relationships that could be construed as a potential conflict of interest.
